# #Coronavirus on TikTok: user engagement with misinformation as a potential threat to public health behavior

**DOI:** 10.1093/jamiaopen/ooad013

**Published:** 2023-02-23

**Authors:** Jonathan D Baghdadi, K C Coffey, Rachael Belcher, James Frisbie, Naeemul Hassan, Danielle Sim, Rena D Malik

**Affiliations:** Division of Genomic Epidemiology and Clinical Outcomes, Department of Epidemiology and Public Health, University of Maryland School of Medicine, Baltimore, Maryland, USA; Division of Genomic Epidemiology and Clinical Outcomes, Department of Epidemiology and Public Health, University of Maryland School of Medicine, Baltimore, Maryland, USA; Division of Urology, Department of Surgery, University of Maryland School of Medicine, Baltimore, Maryland, USA; Division of Urology, Department of Surgery, University of Maryland School of Medicine, Baltimore, Maryland, USA; College of Information Studies, University of Maryland, College Park, Maryland, USA; Philip Merrill College of Journalism, University of Maryland, College Park, Maryland, USA; Division of Urology, Department of Surgery, University of Maryland School of Medicine, Baltimore, Maryland, USA; Division of Urology, Department of Surgery, University of Maryland School of Medicine, Baltimore, Maryland, USA

**Keywords:** TikTok, misinformation, COVID-19, public health

## Abstract

Coronavirus disease (COVID)-related misinformation is prevalent online, including on social media. The purpose of this study was to explore factors associated with user engagement with COVID-related misinformation on the social media platform, TikTok. A sample of TikTok videos associated with the hashtag #coronavirus was downloaded on September 20, 2020. Misinformation was evaluated on a scale (low, medium, and high) using a codebook developed by experts in infectious diseases. Multivariable modeling was used to evaluate factors associated with number of views and presence of user comments indicating intention to change behavior. One hundred and sixty-six TikTok videos were identified and reviewed. Moderate misinformation was present in 36 (22%) videos viewed a median of 6.8 million times (interquartile range [IQR] 3.6–16 million), and high-level misinformation was present in 11 (7%) videos viewed a median of 9.4 million times (IQR 5.1–18 million). After controlling for characteristics and content, videos containing moderate misinformation were less likely to generate a user response indicating intended behavior change. By contrast, videos containing high-level misinformation were less likely to be viewed but demonstrated a nonsignificant trend towards higher engagement among viewers. COVID-related misinformation is less frequently viewed on TikTok but more likely to engage viewers. Public health authorities can combat misinformation on social media by posting informative content of their own.

## INTRODUCTION

Misinformation includes misleading or false information that contradicts scientific consensus.^1^ On social media, misinformation is shared relatively freely among users and spreads farther and faster than the truth.^2–4^ A single false post or story can dominate searches on a given topic.^5^ Even when users identify misinformation in real-time, corrections or rebuttals can register as user engagement that amplifies a story. Thus, misinformation can spread even as it is acknowledged to be wrong. When misinformation that threatens public health spreads across borders, it becomes an infodemic.^6–9^

Exposure to coronavirus disease (COVID)-related misinformation online can influence health-related beliefs, healthcare-seeking behaviors, and mental well-being.^10–13^ On social media, COVID-related misinformation has been documented on Twitter, Facebook, and TikTok.^14–18^ Compared to other platforms, TikTok has a user base that is expanding, particularly among younger, lower income adults living in cities.^19^ Thus, COVID-related content has the potential to reach an audience on TikTok that may not otherwise receive important public health messaging.^20^

Though COVID-related content has been described online and on social media, the response from users is not as well understood.^21^ The purpose of this study was to determine what types of videos on TikTok engage users, elicit user comments, and potentially lead to changes in behavior.

## METHODS

We retrospectively reviewed a random sample of user-created videos on TikTok associated with the hashtag #coronavirus. Videos were eligible for inclusion if published in English on or before the date of the search (September 1, 2020). Conducting the search on a single day was used to capture a “snapshot” of misinformation on social media at a moment in time.^14^ The #coronavirus hashtag was selected because it generated relevant videos with the largest cumulative number of views (97.8 billion) and has been used by other investigators for similar purposes.^16^ Other search terms generated sets of videos with fewer cumulative views, including #covid19 (37.4 billion views), #covid (6.1 billion views), #covid_19 (1.8 billion), and #covid2019 (82.7 million views). Videos were excluded if unviewable due to privacy settings or determined based on preliminary review to be unrelated to COVID-19, related illness, or related public health measures. Preliminary review was used to identify videos that were unrelated to COVID-19 but used the #coronavirus hashtag to boost appearances in search results.

Videos and related metadata were downloaded using a third-party TikTok Scraper (https://github.com/drawrowfly/tiktok-scraper) that is freely available under an MIT license. This tool uses TikTok's Web API to download videos and related metadata.

Prior to review of video content, a codebook was developed by a hospital epidemiologist with expertise in infectious diseases (JB) and a clinician with expertise with social media research (RM). The codebook reflected public health guidance related to COVID-19 and served as a reference to identify misinformation (see Supplemental Material). Videos were viewed and annotated by RB, JF, DS, and RM between September 2020 and January 2021. The first 9 videos were reviewed and annotated by all reviewers, and independent ratings were compared for discrepancies and adjudicated to ensure consistency. Once interrater consistency was above 90%, subsequent videos were reviewed independently.

Prior to review of videos, evidence-based information related to each section of the codebook was reviewed by the research team. Presence of misinformation in each video was graded as low, moderate, or high based on the extent and degree of misinformation. For instance, a video implying that surgical masks were contaminated with COVID-19 because they originated in China was considered highly misinformative. A video implying that the COVID-19 pandemic was predicted on a television show was considered moderately misinformative.

Video quality was assessed using 2 validated scales: the Patient Education Materials Assessment Tool (PEMAT) and DISCERN.^22^^,^^23^ PEMAT is a systematic method available through the Agency for Healthcare Research and Quality to evaluate the *understandability* and *actionability* of patient education materials.^23^ PEMAT understandability and actionability scores were categorized as high (≥80) or low/moderate (<80). DISCERN was designed to help health consumers assess the quality of written information about treatment choices for a health problem.

Video comments were reviewed to identify requests for medical advice, provision of medical advice, reported intention to change behavior, or commercial advertising. Comments reflecting each category were considered present if at least one relevant comment was found. All comments potentially indicating intent to change behavior were reviewed by the research team as a group. Only comments that expressed a clear intention to change behavior (eg, “I will do…”) were included in this category. Comments that expressed mixed intention or uncertainty (eg, “Does anyone know if…?”) or agreement without related behavior (eg, “Your videos are so inspiring”) were not counted as reflecting intent to change behavior.

Multivariable regression with robust standard errors was used to evaluate the association between video characteristics and outcomes. Negative binomial regression was used for count outcomes, while logistic regression was used for binary outcomes. Covariates were included in multivariable models if associated with the outcome of interest on bivariate analysis with a *P*-value < .10 and not highly correlated with other covariates. Covariates related to characteristics of the video publisher were collected from their biography or username. Individuals who self-identified as a physician or doctor were categorized among other official sources. In one instance, a physician was a verified TikTok user. In other cases, these claims could not be verified. Coefficients from negative binomial regression are reported as the incidence rate ratio (IRR), while coefficients from logistic regression are reported as the odds ratio (OR). All coefficients from multivariable models are reported with 95% confidence intervals (95% CI).

## RESULTS

Out of 1000 TikTok videos identified during the initial search, 166 (17%) met the inclusion criteria and were included in the study (Table 1). Videos were published between March 14, 2020 and August 22, 2020. Twenty-six videos (16%) were posted by an official source, including 7 videos from foundations or advocacy groups, 6 from governmental agencies, 3 from doctors, 3 from other healthcare providers, and 1 from a professional society. Seventy-six (46%) videos included humor, and 91 (55%) were set to music. Fifty-five (33%) videos were published by women, and 93 (56%) of publishers were under the age of 30. Only 2 (1.2%) videos were suspected of commercial bias. Videos were viewed a median of 8.4 million times (interquartile range [IQR] 4.9M–18M), and the median number of comments was 6871 (IQR 3348–13 000).

**Table 1.** Video views by characteristic and content

Video characteristics	Videos (%)	Median views (IQR)
	*n* = 166	
Official publisher		
Healthcare worker	8 (5)	14M (13–14M)
Foundation/government	13 (4)	28M (19–35M)
News media/outlet	4 (2)	7.2M (5.2–9.2M)
Any official source^a^	26 (16)	23M (10–35M)
Unofficial publisher		
Patient	5 (3)	18M (14–21M)
Other	136 (82)	7.4M (4.4–1M)
Subject content		
Risk factors^a^	22 (13)	21M (13M–35M)
Symptoms^a^	26 (16)	16M (7.7M–37M)
Modes of transmission^b^	38 (23)	12M (6.4M–24M)
Prevention^a^	90 (54)	11M (5.6M–23M)
Content related to prevention		
Masks	60 (36)	9.5M (5.6M–22M)
Hand hygiene	53 (32)	8.4M (4.9M–27M)
Distancing	45 (27)	7.7M (4.7M–14M)
Quarantine/isolation^a^	35 (21)	5.0M (3.4M–11M)
Limiting exposures	51 (30)	9.5M (4.9M–23M)
Content related to masks		
Demonstration of use^b^	43 (26)	10M (7.1M–24M)
Positive sentiment	37 (22)	8.1M (5–18M)
Neutral sentiment	20 (12)	10M (7.3–37M)
Negative sentiment	3 (2)	5.0M (4.7–21M)
Content related to testing		
Testing discussed^b^	13 (8)	5.0M (3.7M–7.7M)
Positive sentiment	9 (5)	5.0M (3.7–7.7M)
Neutral sentiment	7 (4)	8.7M (5.1–20M)
Negative sentiment	0 (0)	–
Misinformation		
Absent	119 (72)	8.5M (5.3–19M)
Moderate	36 (22)	6.8M (3.6–16M)
High	11 (7)	9.4M (5.1–18M)
DISCERN rating		
Low^b^	50 (30)	9.9M (4.5–22M)
Moderate	84 (51)	9.9M (4.5M–22M)
High	32 (19)	8.2M (5.0M–15M)
PEMAT rating		
High understandability^b^	110 (66)	9.4M (5.4–21M)
High actionability	15 (9)	26M (5.6M–35M)
Total	166	8.4M (4.9M–18M)

Abbreviation: PEMAT: Patient Education Materials Assessment Tool.

a Significantly different than comparator group at an *α* < .001.

b Significantly different than comparator group with *P*-value < .05.

**Figure 1.**
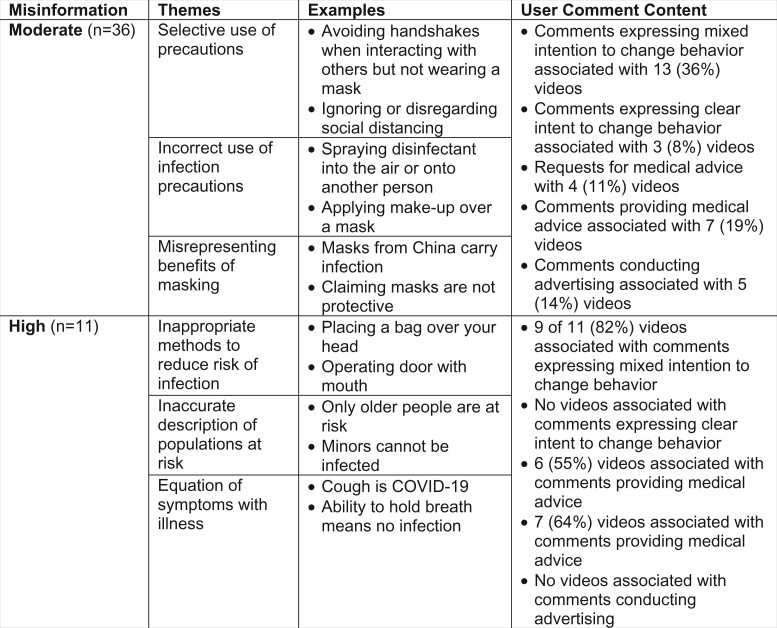
User response to TikTok videos based on level of misinformation.

Forty-seven (28%) videos contained misinformation, including 36 videos with a moderate level of misinformation and 11 videos with a high level of misinformation. Videos containing moderate misinformation were viewed a median of 6.8 million times (IQR 3.6–16 million), and videos containing high-level misinformation were viewed a median of 9.4 million times (IQR 5.1–18 million). Examples of high- and moderate-level misinformation are included in a joint display with comment content in Figure 1. Examples of misinformation included claims that recommendations for quarantine were the result of a conspiracy and that masks do not offer protection against infection.

Results from multivariable modeling to identify factors independently associated with how many times a video was viewed are listed in Table 2. Videos containing misinformation were viewed a median of 7.4M times (IQR 3.6M–18.1M). After controlling for video subject matter, videos published by an official source (IRR 1.49; 95% CI 1.09–2.05), such as a government agency, foundation, or advocacy group, were more likely to be viewed. View counts were significantly lower among videos suspected of commercial bias (IRR 0.23; 95% CI 0.15–0.35) and videos containing a high level of misinformation (IRR 0.59; 95% CI 0.39–0.90).

**Table 2.** Factors associated with view counts and comments indicating intention to change behavior on TikTok videos with the #Coronavirus hashtag

Factor	View count^a^	Behavior change^b^
	IRR (95% CI)	OR (95% CI)
Predictor of interest		
Moderate misinformation	0.78 (0.57–1.05)	**0.25 (0.078–0.77)**
High misinformation	**0.59 (0.39–0.90)**	6.14 (0.72–52.6)
Publisher characteristics		
Publisher age < 30	**0.57 (0.45–0.73)**	0.40 (0.14–1.11)
Official source^c^	**1.49 (1.09–2.05)**	0.35 (0.045–2.71)
Video content		
Inclusion of humor	1.20 (0.93–1.55)	0.487 (0.18–1.28)
Content related to symptoms	**1.72 (1.25–2.37)**	3.18 (0.92–10.9)
Content related to testing	0.96 (0.61–1.50)	**10.1 (2.25–45.5)**
Content related to prevention	**1.66 (1.31–2.09)**	**10.2 (3.46–29.8)**
Content related to quarantine	**0.50 (0.39–0.64)**	**0.14 (0.048–0.40)**
Commercial bias	**0.23 (0.15–0.35)**	*-omitted-*
Video quality		
DISCERN rating of moderate-high	**0.67 (0.51–0.89)**	2.07 (0.66–6.46)
PEMAT understandability high	1.18 (0.92–1.50)	**3.22 (1.24–8.34**)
PEMAT actionability high	0.97 (0.64–1.46)	1.65 (0.14–19.4)

Bold values represent factors that were significant at a threshold of α < .05.

a View count was modeled using multivariable negative binomial regression.

b Behavior change was evaluated based on user comments reporting intention to change behavior and was modeled using multivariable logistic regression.

c Government, foundation, or advocacy group.

User comments indicating intention to change behavior were associated with 117 videos (70.5%), including 25 videos containing misinformation (15.1%). After controlling for video subject matter, user-reported intention to change behavior was associated with high PEMAT understandability (OR 3.22; 95% CI 1.24–8.34). Moderate misinformation (OR 0.25; 95% CI 0.078–0.77) was associated with decreased likelihood of user-reported intention to change behavior. High-level misinformation was associated with a nonsignificant trend towards increased likelihood of user engagement in terms of behavior change (OR 6.14; 95% CI 0.72–52.6).

## DISCUSSION

In this retrospective review of early COVID-related content on the social media platform TikTok, 28% of videos contained at least moderate-level misinformation. The relationship between misinformation and user engagement was complex. Videos containing high-level misinformation were less likely to be viewed but more likely to engage viewers. On the other hand, videos containing moderate misinformation were viewed as frequently as videos without misinformation but were less engaging.

Previous research has identified that COVID misinformation on TikTok is highly viewed.^24^ Our study builds on this literature by examining user comments posted in response to misinformative videos. The experience of users on TikTok can include passive consumption of content or active interaction with other users.^25^ By differentiating videos based on the extent and degree of misinformation, we were able to explore further how misinformation provokes active engagement.

Misinformation spreads more easily than true information, and public health misinformation spreads more quickly when public health experts express uncertainty.^3^^,^^26^ In this study, though videos containing high-level misinformation were less likely to be viewed, misinformative content was nonetheless viewed millions of times. One in 4 misinformative videos elicited comments indicating intention to change behavior. Though misinformative videos were often humorous and frequently ridiculous, at least some users took them seriously. In the context of a coordinated public health response, those instances were troubling.

Behaviors spread on social media through social reinforcement.^2^^,^^27^^,^^28^ Because we were unable to measure user behavior directly, we represented behavior using proxy outcomes: view counts and user comments reporting intention to change behavior. Public health agencies should take note that videos posted by an official source were more likely to be viewed, and highly understandable videos were more likely to generate a user response indicating intended behavior change. Clear, understandable, and informative videos posted by trustworthy sources may be able to counteract the negative impact of misinformation on TikTok. To facilitate dissemination of informative content, authoritative sources may consider partnering with organizations that are better equipped or more experienced communicating with the public, such as mass media.^4^

This study had several limitations. First, the videos included in our analysis represent a sample of public English-language videos associated with the hashtag #coronavirus identified on the day we conducted the search. Our study is subject to potential bias that may have been introduced if the TikTok algorithm selectively displayed or suppressed specific videos. Second, our study was limited to videos in English despite COVID-19 related rumors, stigma, and conspiracy theories have been posted in 25 languages from 87 countries.^29^ Third, we did not examine the characteristics of users posting comments, which are known to influence susceptibility to health-related misinformation.^12^ Thus, we were unable to understand how viewer characteristics might interact with video characteristics to affect engagement. Finally, because we used human reviewers to identify misinformation, our work may be less scalable than research based on automated algorithms.^30^

In conclusion, the relationship between misinformation on TikTok and user engagement is complex. For a niche audience, misinformative content may be highly engaging. To combat misinformation, public health agencies should work to establish more of a presence on TikTok and other social media platforms.

## Supplementary Material

ooad013_Supplementary_Data

## Data Availability

The data underlying this article are available on Dryad (https://doi.org/10.5061/dryad.bvq83bkdp).
