# Supplementary material for: #Coronavirus on TikTok: user engagement with misinformation as a potential threat to public health behavior
Source: JAMIA Open. 2023 Feb 23;6(1):ooad013. doi: 10.1093/jamiaopen/ooad013 (PMC9949581; doi:10.1093/jamiaopen/ooad013)
Supplement: ooad013_Supplementary_Data [file ooad013_supplementary_data.docx]

Suppl. Material – Codebook used to review COVID-related videos on TikTok

| **ITEM** | **ITEM/RESPONSE DESCRIPTION & NOTES** |  | **RESPONSE** | **VALUE** |
| --- | --- | --- | --- | --- |
| **Link** | *URl for the post* |  |  |  |
| **Date** | *Date of search* |  |  | *Pre-filled* |
| **Views** | *How many times the post has been viewed on TikTok as of day of search?* |  |  | *Pre-filled* |
| **Likes** | *How many likes did the post get on TikTok as of the day of the search?* |  |  | *Pre-filled* |
| **# Comments** | *How many comments did the post get on TikTok as of the date of the search?* |  |  | *Pre-filled* |
| **Reviewer** | *Fill in your initials* |  |  | *Fill in* |
| **Language** | *Is the post in English? (STOP IF NOT IN ENGLISH)* |  | Yes | *0* |
|  |  |  | No --> exclude | *1* |
|  |  |  | N/A (no language) | *2* |
| **Privacy** | *Is it private settings? (STOP IF PRIVATE)* |  | Yes --> exclude | *0* |
|  |  |  | No | *1* |
| **Music** | *Is there music in the post?* |  | Yes | *0* |
|  |  |  | No | *1* |
|  |  |  | N/A | *2* |
| **Hashtags** | *Please copy/paste the string of hashtags used in the post* |  | Free text | *Fill in* |
| **PUBLISHER TYPE** | *What type of user posted the content?* |  | Patient | **0** |
|  |  |  | Family Member | **1** |
|  |  |  | Other Layperson User/Unclear | **2** |
|  |  |  | Doctor | **3** |
|  |  |  | Other healthcare professional | **4** |
|  |  |  | Hospital/clinic | **5** |
|  |  |  | University | **6** |
|  |  |  | Professional Society | **7** |
|  |  |  | Foundation/advocacy group | **8** |
|  |  |  | Governmental | **9** |
|  |  |  | News source/media outlet | **10** |
|  |  |  | Commercial media/industry | **11** |
|  |  |  | Health/wellness channel | **12** |
|  |  |  | Academic journal/publisher | **13** |
|  |  |  | Medical education | **14** |
|  |  |  | Other/unclear | **15** |
| **GENDER OF PUBLISHER** | *What is the gender of the user who posted the TikTok?* |  | Male | **0** |
|  |  |  | Female | **1** |
|  |  |  | N/A,Not an individual human | **2** |
|  |  |  | Other/unclear | **3** |
| **AGE GROUP OF PUBLISHER** | *What is the age of the user posting the TikTok?* |  | <30 | *0* |
|  |  |  | 30-50 | *1* |
|  |  |  | >50 | *2* |
|  |  |  | N/A not an individual human | *3* |
|  |  |  | Unclear/cannot discern | *4* |
| **PUBLISHER #FOLLOWERS** | *How many followers does the user posting the TikTok have?* |  | (numerical) |  |
| **PUBLISHER #LIKES** | *How many likes does the user posting the TikTok have?* |  | (numerical) |  |
| **MAIN TOPIC** |  |  | Clinical disease | **0** |
|  |  |  | Epidemiology | **1** |
|  |  |  | Infection prevention | **2** |
|  |  |  | Treatment | **3** |
|  |  |  | Testing | **4** |
|  |  |  | Other | **5** |
| **DESCRIPTION OF RISK FACTORS** | *Does the video describe risk factors for getting COVID-19 (i.e. male gender, age, comorbidities)* |  | Yes | *0* |
|  |  |  | No | *1* |
| **DESCRIPTION OF SYMPTOMS** | *Does the video describe COVID-19 symptoms?* |  | Yes | *0* |
|  |  |  | No | *1* |
| **COMPARISON TO INFLUENZA** | *Does the video compare COVID-19 to influenza?* |  | Yes | *0* |
|  |  |  | No | *1* |
| **MODES OF TRANSMISSION** | *Does the video discuss COVID-19 transmission?* |  | Yes | *0* |
|  |  |  | No | *1* |
| **ASYMPTOMATIC TRANSMISSION** |  |  | Yes | *0* |
|  |  |  | No | *1* |
|  |  |  | N/A | *2* |
| **PREVALENCE IN THE COMMUNITY** |  |  | Yes | *0* |
|  |  |  | No | *1* |
| **INFLUENCE OF WEATHER/SEASONALITY** | *Does the video discuss the influence of weather/seasonality on COVID-19 transmission?* |  | Yes | *0* |
|  |  |  | No | *1* |
| **MASKS** | *Does the video discuss the use of masks?* |  | Yes | *0* |
|  |  |  | No | *1* |
| **MASK SENTIMENT** | *What is the sentiment regarding masks in the video?* |  | Positive | **0** |
|  |  |  | Negative | **1** |
|  |  |  | Neutral/Unclear | **2** |
|  |  |  | N/A | **3** |
| **DEMONSTRATE WEARING MASK** | *Does the video describe how to wear a mask?* |  | Yes | *0* |
|  |  |  | No | *1* |
|  |  |  | N/A | *2* |
| **MASK WORM CORRECTLY** | *If decribed or shown are masks worn correctly (covering mouth and nose and chin)?* |  | Yes | *0* |
|  |  |  | No | *1* |
|  |  |  | N/A | *2* |
| **N95** | *Do they discuss/show N95 masks?* |  | Yes | *0* |
|  |  |  | No | *1* |
|  |  |  | N/A | *2* |
| **SURGICAL MASK** | *Do they discuss/show surgical masks?* |  | Yes | *0* |
|  |  |  | No | *1* |
|  |  |  | N/A | *2* |
| **FACE COVERING/CLOTH MASK** | *Do they discuss/show face coverings or cloth masks?* |  | Yes | *0* |
|  |  |  | No | *1* |
|  |  |  | N/A | *2* |
| **EYE PROTECTION** |  |  | Yes | *0* |
|  |  |  | No | *1* |
| **HAND HYGIENE** |  |  | Yes | *0* |
|  |  |  | No | *1* |
| **SOCIAL DISTANCING** |  |  | Yes | *0* |
|  |  |  | No | *1* |
| **TESTING** | *Do they mention testing for COVID-19 in the video?* |  | Yes | *0* |
|  |  |  | No | *1* |
| **TESTING SENTIMENT** | *What is the sentiment regarding testing in the video?* |  | Positive | **0** |
|  |  |  | Negative | **1** |
|  |  |  | Neutral/Unclear | **2** |
|  |  |  | N/A | **3** |
| **TREATMENT** | *Do they mention treatment for COVID-19 in the video?* |  | Yes | *0* |
|  |  |  | No | *1* |
| **EVIDENCE-BASED TREATMENT** | *Do they mention evidence-based treatment (i.e. Remdesivir, dexamethasone, medication in context of a trial)?* |  | Yes | *0* |
|  |  |  | No | *1* |
|  |  |  | N/A | *2* |
| **NON EVIDENCE-BASED TREATMENT** | *Do they mention non-evidence-based medicine (i.e. Hydroxychloroquine, Hydroxychloroquine + azithromycin)?* |  | Yes | *0* |
|  |  |  | No | *1* |
|  |  |  | N/A | *2* |
| **ALTERNATIVE TREATENT** | *Are the aims clear?* |  | Yes | *0* |
|  |  |  | No | *1* |
|  |  |  | N/A | *2* |
| **PREVENTION DISCUSSED** |  |  | Yes | *0* |
|  |  |  | No | *1* |
| **TYPE OF PREVENTION** | *Good Preventative Measures – social distancing, hand hygiene, masks, quaranting, limiting exposures, not touching your face* |  | Accurate | **0** |
|  | *Inaccurate – anything else (i.e. eating foods, zinc, vit D)* |  | Inaccurate | **1** |
| **SOCIAL DISTANCING** |  |  | Yes | *0* |
|  |  |  | No | *1* |
| **HAND HYGEINE** |  |  | Yes | *0* |
|  |  |  | No | *1* |
| **QUARANTINING** |  |  | Yes | *0* |
|  |  |  | No | *1* |
| **LIMITING EXPOSURES** |  |  | Yes | *0* |
|  |  |  | No | *1* |
| **NOT TOUCHING YOUR FACE** |  |  | Yes | *0* |
|  |  |  | No | *1* |
| **RACIAL DISPARITIES DISCUSSED IN POST?** | *Did the post discuss racial disparities in covid-19?* |  | No | **0** |
|  |  |  | Yes | **1** |
| **SETTING OF THE VIDEO?** | *Where was the video filmed?* |  | Social setting (e.g., party) | *0* |
|  |  |  | Clinical Setting (e.g. hospital) | *1* |
|  |  |  | Office setting (e.g. at desk) | *2* |
|  |  |  | Home setting (e.g., kitchen) | *3* |
|  |  |  | Unclear (can't really tell) | *4* |
|  |  |  | N/A no setting- (e.g. just text) | *5* |
| **FREE TEXT ABOUT CONTENT** | *Free text if you wish to specify further about type of content* |  | **Free text or* ***N/A if none*** 🡪 *DO NOT LEAVE BLANK* | *Fill in* |
| **DISCERN 1: are the aims clear?** | Are the aims clear? |  | No | **1** |
|  |  |  | *Blank* | **2** |
|  |  |  | Partially | **3** |
|  |  |  | *Blank* | **4** |
|  |  |  | Yes | **5** |
| **DISCERN 2: does it achieve its aims?** | Does it achieve its aims? |  | No | **1** |
|  |  |  | *Blank* | **2** |
|  |  |  | Partially | **3** |
|  |  |  | *Blank* | **4** |
|  |  |  | Yes | **5** |
| **DISCERN 3: is it relevant?** | Is it relevant? |  | No | **1** |
|  |  |  | *Blank* | **2** |
|  |  |  | Partially | **3** |
|  |  |  | *Blank* | **4** |
|  |  |  | Yes | **5** |
| **DISCERN 4: is it clear what sources of information were used to compile the video?** | Is it clear what sources of information were used to compile the post (other than the author or producer)? |  | No | **1** |
|  |  |  | *Blank* | **2** |
|  |  |  | Partially | **3** |
|  |  |  | *Blank* | **4** |
|  |  |  | Yes | **5** |
| **DISCERN 5: is it clear WHEN the information/sources used to make the video were from?** | Is it clear WHEN the information/sources used to make the video were from? |  | No | **1** |
|  |  |  | *Blank* | **2** |
|  | [Is it clear when the information used or reported in the publication was produced?] |  | Partially | **3** |
|  |  |  | *Blank* | **4** |
|  |  |  | Yes | **5** |
| **DISCERN 6: is it balanced and unbiased?** | Is it balanced and unbiased? |  | No | **1** |
|  |  |  | *Blank* | **2** |
|  |  |  | Partially | **3** |
|  |  |  | *Blank* | **4** |
|  |  |  | Yes | **5** |
| **DISCERN 7: does it provide details of additional sources of support and information?** | Does it provide details of additional sources of support and information? |  | No | **1** |
|  |  |  | *Blank* | **2** |
|  |  |  | Partially | **3** |
|  |  |  | *Blank* | **4** |
|  |  |  | Yes | **5** |
| **DISCERN 8: does it refer to areas of uncertainty** | Does it refer to areas of uncertainty? |  | No | **1** |
|  |  |  | *Blank* | **2** |
|  |  |  | Partially | **3** |
|  |  |  | *Blank* | **4** |
|  |  |  | Yes | **5** |
| **DISCERN 9: Does it describe how testing/each treatment works?** | Does it describe how testing/each treatment works? |  | No | **1** |
|  |  |  | *Blank* | **2** |
| **for videos about testing/treatment* |  |  | Partially | **3** |
| *[otherwise leave blank)* |  |  | *Blank* | **4** |
|  |  |  | Yes | **5** |
| **DISCERN 10: Does it describe the BENEFITS of testing/each treatment?** | Does it describe the benefits of testing/ each treatment? |  | No | **1** |
|  |  |  | *Blank* | **2** |
| **for videos about testing/treatment* | HINT: Benefits can include identifying asymptomatic transmisison, controlling or getting rid of symptoms, preventing morbidity |  | Partially | **3** |
| *[otherwise leave blank)* |  |  | *Blank* | **4** |
|  |  |  | Yes | **5** |
| **DISCERN 11: Does it describe the RISKS of testing/each EB - treatment?** | Does it describe the risks of testing / each treatment? |  | No | **1** |
|  |  |  | *Blank* | **2** |
| **for videos about testing/treatment* | HINT: Risks can include side effects, complications and adverse reactions to treatment, both short-term and long term. |  | Partially | **3** |
| *[otherwise leave blank)* |  |  | *Blank* | **4** |
|  |  |  | Yes | **5** |
| **DISCERN 12: Does it describe what would happen if no screening/treatment is used?** | Does it describe what would happen if no screening/treatment is used? |  | No | **1** |
|  |  |  | *Blank* | **2** |
| **for videos about screening/treatment* |  |  | Partially | **3** |
| *[otherwise leave blank)* |  |  | *Blank* | **4** |
|  |  |  | Yes | **5** |
| **DISCERN 13: Does it describe how the treatment choices affect overall quality of life?** | Does it describe how the screening/treatment choices affect overall quality of life? |  | No | **1** |
|  |  |  | *Blank* | **2** |
| **for videos about screening/treatment* |  |  | Partially | **3** |
| *[otherwise leave blank)* |  |  | *Blank* | **4** |
|  |  |  | Yes | **5** |
| **DISCERN 14: Is it clear that there may be more than one possible screening/treatment choice?** | Is it clear that there may be more than one possible screening/treatment choice? |  | No | **1** |
|  |  |  | *Blank* | **2** |
|  |  |  | Partially | **3** |
|  |  |  | *Blank* | **4** |
|  |  |  | Yes | **5** |
| **DISCERN 15: Does it provide support for shared decision-making? (omit)** | Does it provide support for shared decision-making? |  | No | **1** |
|  |  |  | *Blank* | **2** |
|  | HINT Look for suggestions of things to discuss with family, friends, doctors or other health professionals concerning treatment choices. |  | Partially | **3** |
|  |  |  | *Blank* | **4** |
|  |  |  | Yes | **5** |
| **DISCERN 16 (overall rating)** | Based on the answers to all of the above questions, rate the overall quality of the publication as a source of information about treatment choices [the topic] |  | Low [*Serious or extensive shortcomings*] | **1** |
|  |  |  | *Blank* | **2** |
|  |  |  | Moderate [*Potentially important but not serious shortcomings*] | **3** |
|  |  |  | *Blank* | **4** |
|  |  |  | High [*minimal shortcomings*] | **5** |
| **MISINFORMATION** | *How much misinformation is present 🡪 comparison to CDC* |  | None | **1** |
|  |  |  | *Blank* | **2** |
|  |  |  | Moderate | **3** |
|  |  |  | *Blank* | **4** |
|  |  |  | High | **5** |
| **TYPE OF MISINFORMATION** | *What was the misinformation about (if none, please type N/A)?* |  | Free text (or N/A) |  |
| **COMMERCIAL BIAS (anywhere in the video)?** | *Does there appear to be commercial bias/conflict of interest anywhere in the video?* |  | No | **0** |
|  |  |  | Yes | **1** |
|  |  |  | Unknown | **2** |
| **TYPE OF COMMERCIAL CONTENT** | What were they advertising/promoting? (if none, type N/A) |  | Free text (or N/A) |  |
| **PEMAT 1 (Understandability: Content)** | The material makes its purpose completely evident |  | Disagree | **0** |
|  |  |  | Agree | **1** |
| **PEMAT 3 (Understandability: Word Choice & Style)** | The material uses common, everyday language. |  | Disagree | **0** |
|  |  |  | Agree | **1** |
| **PEMAT 4 (Understandability: Word Choice & Style)** | Medical terms are used only to familiarize audience with the terms. When used, medical terms are defined. |  | Disagree | **0** |
|  |  |  | Agree | **1** |
| **PEMAT 5 (Understandability: Word Choice & Style)** | The material uses the active voice. |  | Disagree | **0** |
|  |  |  | Agree | **1** |
| **PEMAT 12 (Understandability: Layout & Design)** | The material uses visual cues (e.g., arrows, boxes, bullets, bold, larger font, highlighting) to draw attention to key points |  | Disagree | **0** |
|  |  |  | Agree | **1** |
|  |  |  | Video – N/A | **2** |
| **PEMAT 13 (Understandability: Layout & Design)** | Text on the screen is easy to read. |  | Disagree | **0** |
|  |  |  | Agree | **1** |
|  |  |  | No text or all text is narrated – N/A | **2** |
| **PEMAT 14 (Understandability: Layout & Design)** | The material allows the user to hear the words clearly (e.g., not too fast, not garbled). |  | Disagree | **0** |
|  |  |  | Agree | **1** |
|  |  |  | No narration – N/A | **2** |
| **PEMAT 18 (Understandability: Use of Visual Aids)** | The material uses illustrations and photographs that are clear and uncluttered. |  | Disagree | **0** |
|  |  |  | Agree | **1** |
|  |  |  | No visual aids – N/A | **2** |
| **PEMAT 20 (Actionability)** | The material clearly identifies at least one action the user can take. |  | Disagree | **0** |
|  |  |  | Agree | **1** |
| **PEMAT 21 (Actionability)** | The material addresses the user directly when describing actions. |  | Disagree | **0** |
|  |  |  | Agree | **1** |
| **PEMAT 22 (Actionability)** | The material breaks down any action into manageable, explicit steps. |  | Disagree | **0** |
|  |  |  | Agree | **1** |
| **PEMAT 25 (Actionability)** | The material explains how to use the charts, graphs, tables, or diagrams to take actions. |  | Disagree | **0** |
|  |  |  | Agree | **1** |
|  |  |  | No charts/graphs/tables/diagrams – N/A | **2** |
|  |  |  |  |  |
| **PEMAT UNDERSTANDABILITY TOTAL** | *Score for items 1-19 [hidden]* |  | *Sum total points → divide by total number of items excluding N/A items → multiply results by 100 (this will yield the % score)* | *Fill in* |
| **PEMAT ACTIONABILITY TOTAL** | *Score for items 20-26 [hidden]* |  | *Sum total points → divide by total number of items excluding N/A items → multiply results by 100 (this will yield the % score)* | *Fill in* |
| **BEHAVIOR CHANGE (reflected in comments)?** | *Evidence that the video has influenced viewers based on comments?* |  | No influence | **0** |
|  |  |  | Yes influence | **1** |
|  |  |  | Mixed response (no and yes) | **2** |
|  |  |  | Unknown/no comments | **3** |
| **REQUESTING MEDICAL ADVICE IN COMMENTS?** | *Do any of the comments request medical advice?* |  | No | **0** |
|  |  |  | Yes | **1** |
|  |  |  | N/A | **2** |
| **MEDICAL ADVICE GIVEN (comment section)** | *Do any of the comments give medical advice?* |  | No advice given | **0** |
|  |  |  | Advice refused/referred to doctor | **1** |
|  |  |  | Some advice given | **2** |
|  |  |  | N/A | **3** |
| **ADVERTISING IN COMMENTS** | *Is there any commercial advertising in the comments? (e.g. buy my special formula? buy my book?)* |  | No | **0** |
|  |  |  | Yes | **1** |
|  |  |  | N/A | **2** |
| **INTERESTING COMMENTS/QUOTES** | *If any comments include the areas mentioned above and are interesting/noteworthy, please copy/paste (or N/A if none)* |  | Free text (or N/A if none) | *Fill in* |
| **NOTES** | *Anything about the record worth documenting* |  | *Free text (or N/A if none)* | *Fill in* |
